# Experimentally Infected Domestic Ducks Show Efficient Transmission of Indonesian H5N1 Highly Pathogenic Avian Influenza Virus, but Lack Persistent Viral Shedding

**DOI:** 10.1371/journal.pone.0083417

**Published:** 2014-01-02

**Authors:** Hendra Wibawa, John Bingham, Harimurti Nuradji, Sue Lowther, Jean Payne, Jenni Harper, Akhmad Junaidi, Deborah Middleton, Joanne Meers

**Affiliations:** 1 Commonwealth Scientific and Industrial Research Organisation (CSIRO), Australian Animal Health Laboratory, Geelong, Australia; 2 School of Veterinary Science, The University of Queensland, Brisbane, Australia; 3 Disease Investigation Centre Regional IV Wates, Yogyakarta, Indonesia; 4 Indonesian Research Center for Veterinary Science, Bogor, West Java, Indonesia; Centers for Disease Control and Prevention, United States of America

## Abstract

Ducks are important maintenance hosts for avian influenza, including H5N1 highly pathogenic avian influenza viruses. A previous study indicated that persistence of H5N1 viruses in ducks after the development of humoral immunity may drive viral evolution following immune selection. As H5N1 HPAI is endemic in Indonesia, this mechanism may be important in understanding H5N1 evolution in that region. To determine the capability of domestic ducks to maintain prolonged shedding of Indonesian clade 2.1 H5N1 virus, two groups of Pekin ducks were inoculated through the eyes, nostrils and oropharynx and viral shedding and transmission investigated. Inoculated ducks (n = 15), which were mostly asymptomatic, shed infectious virus from the oral route from 1 to 8 days post inoculation, and from the cloacal route from 2–8 dpi. Viral ribonucleic acid was detected from 1–15 days post inoculation from the oral route and 1–24 days post inoculation from the cloacal route (cycle threshold <40). Most ducks seroconverted in a range of serological tests by 15 days post inoculation. Virus was efficiently transmitted during acute infection (5 inoculation-infected to all 5 contact ducks). However, no evidence for transmission, as determined by seroconversion and viral shedding, was found between an inoculation-infected group (n = 10) and contact ducks (n = 9) when the two groups only had contact after 10 days post inoculation. Clinical disease was more frequent and more severe in contact-infected (2 of 5) than inoculation-infected ducks (1 of 15). We conclude that Indonesian clade 2.1 H5N1 highly pathogenic avian influenza virus does not persist in individual ducks after acute infection.

## Introduction

Although an outbreak of highly pathogenic avian influenza (HPAI) in poultry due to H5N1 virus was first reported in 1959 [1], only the A/goose/Guangdong/1/96 lineage H5N1 viruses have spread widely and have persisted over time. Since the first isolation of the progenitor virus in southern China in 1996 [2], this “Eurasian H5N1 HPAI” virus lineage has spread to over 60 countries throughout Asia and into Europe and Africa [3] and has continued to circulate for more than 16 years. These viruses continue to evolve via mutation and genetic reassortment with other avian influenza (AI) viruses, resulting in multiple virus genotypes and geographically related sublineages [4], [5]. Most H5N1 HPAI virus outbreaks have occurred in domestic poultry, either in backyard or small commercial farms, indicative of the high incidence rate in these species and resulting in the death or forced culling of more than 400 million domestic poultry [6]. Although H5N1 HPAI viruses have not acquired efficient transmission among people, direct transmission of virus from poultry to humans has caused severe disease and death of 375 people from 630 confirmed cases [7]. Thus, these viruses pose a major challenge for both veterinary and human public health.

The role of wild birds in the transmission and spread of the Eurasian lineage of H5N1 HPAI viruses remains controversial [8]–[10]. Both domestic and wild birds, including migratory waterfowl, free-range village poultry, poultry sold through live bird markets and fighting cocks are likely to be involved in the spread of H5N1 HPAI virus [3], [9], [11]. Difficulties in controlling local and regional movement of poultry and their products, problems in handling the trade (particularly illegal) of live birds, and limited participation of poultry farmers in control strategies are considered as significant factors contributing to the H5N1 HPAI virus epidemic [8], [9], [12]. In Asia, backyard farms are a common feature in villages, where biosecurity measures are inadequately employed, access to veterinary services is often limited [13], [14] and chickens and waterfowl, including domestic ducks, are commonly raised together [15]–[17]. Ducks, particularly mallard-type breeds, are considered central to the maintenance and transmission of H5N1 HPAI viruses because they can replicate these viruses without suffering clinical disease [18]–[21]. Previous studies indicate that domestic ducks are a likely source of H5N1 HPAI viral infection to chickens in smallholder duck farms in Indonesia and husbandry practices of ducks within villages could increase the risk for H5N1 HPAI [15], [22]. In addition, natural reassortment between different AI virus subtypes and endemic H5N1 HPAI viruses can occur in domestic ducks, leading to recurrent interspecies transmission and genetic drift [5]. Preventing transmission events of H5N1 HPAI virus from or into ducks is a key factor in minimizing HPAI virus spread. Therefore, gaining more knowledge on the patterns of H5N1 HPAI virus transmission in this species will assist efforts to control the disease.

Previous studies showed that experimentally infected ducks could shed low pathogenic avian influenza (LPAI) virus for up to 18–20 days post inoculation [23]–[25], while most H5N1 HPAI viruses have been reported to be shed by ducks for only 2–5 days post inoculation [20], [26]–[29]. However, more persistent H5N1 HPAI virus shedding was shown by Hulse-Post *et al.* [21] indicating that mallard ducks can shed this virus up to 17 days post inoculation, despite seroconversion with a significant titer of hemagglutinating antibodies. They found that in sera taken from extended infections, the neutralizing titer against the inoculated virus was higher than against the virus isolated at the last day of shedding. These researchers hypothesized that because H5N1 HPAI viruses are less pathogenic to ducks, they are able to evolve rapidly in ducks through continuous selection of immunological escape mutants within a host. In this hypothesis, as the duck host develops neutralizing antibodies against the challenge virus, new antigenic mutant viruses arise that are not neutralized by the antibody response, and the host is therefore able to maintain the infection after the immune response develops. The implications of this concept are profound: if this occurs widely, then ducks may be the principal drivers for H5N1 HPAI virus evolution. They could remain inapparently infectious for longer than previously recognized, amplifying their infection risk for other susceptible populations. Such long-term infections of H5N1 HPAI virus in ducks have not been described elsewhere. In the study described here, we investigate the importance of this concept in domestic ducks for an H5N1 HPAI virus from Indonesia.

Our previous studies indicated that shedding of Indonesian H5N1 HPAI viruses in Pekin ducks occurred over only a short period, between 1 and 8 days post inoculation, with viral decline coinciding with the development of antibodies [28], [30]. Shedding of virus after this period was not detected by the conventional methods (virus isolation of swab media). To our knowledge, there are no studies that have examined methodically the viral infection and shedding after the acute infection stage in ducks. Also, there have been few comparisons between the shedding patterns of infectious virus and viral RNA in H5N1 HPAI animal models. Therefore, in the present study we attempted to investigate further the importance of extended infection and shedding in Pekin ducks after recovery from H5N1 HPAI infection, by detecting not only presence of virus in swabs but also through detecting transmission to contact ducks. A comprehensive analysis was conducted comparing the shedding of infectious virus and viral RNA from the oral and cloacal routes through the acute infection and post-acute infection periods. The results showed that shedding of infectious virus occurred over a relatively short period for 1–8 days post inoculation (dpi) and viral transmission was not detected after 10 dpi. Shedding of viral RNA was detected over a much longer period. We discuss the implications of our findings for understanding the transmission biology and evolution of H5N1 HPAI virus in ducks and for the interpretation of viral RNA detection for diagnostic purposes.

## Materials and Methods

### Ethics Statement

All birds were handled and cared for in accordance with the animal welfare standard operating procedures of CSIRO-Australian Animal Health Laboratory (AAHL), Geelong, Australia, which are based on the recommendations in the National Health and Medical Research Council's Australian Code of Practice for the Care and Use of Animals for Scientific Purposes. The experimental procedures were conducted with the approval of AAHL's Animal Ethics Committee (number 1377).

### Animals and Bio-containment

Twenty-nine 4 week-old Pekin ducks were used in this study. Before the inoculation date, each duck was given a unique numbered leg band. Ducks were divided into four groups by random assignment and housed in separate rooms at microbiological physical containment level 3 at AAHL with husbandry procedures as described previously [28].

### Virus

An Indonesian H5N1 HPAI clade 2.1 virus, A/duck/BBVW-1003-34368/2007 (IDN 34368), was used in this study. This virus strain had been passaged, from the original duck specimen, three times in embryonated chicken eggs (specific-pathogen free) to obtain the working stock virus. The stock virus was diluted 1∶10 in sterile phosphate buffer saline (PBS), then inoculated at a total volume of 0.5 ml into each duck in the inoculated groups through pathways considered closest to the natural route: 1–2 drops of inoculum into each eye and nostril, and the remainder instilled into the mouth. A back titration established that the inoculum contained 10^7.7^ EID_50_/0.1 ml, which approximately equates to 10^8.4^ EID_50_ in the 0.5 ml administered to each duck.

### Study Design

Two groups of ducks (group 1, n = 5 and group 3, n = 10) were inoculated with the virus and kept in two separate rooms. Two other groups, group 2 (n = 5) and group 4 (n = 9) were contact ducks for inoculated groups 1 and 3, respectively. Our previous study demonstrated that Pekin ducks experimentally infected with this clade 2.1.1 virus showed subclinical acute infection that occurred between 1–8 dpi, as determined by detection of infectious virus in tissues and in oral swabs [30]. To investigate virus transmission during the acute infection stage, group 2 (contact ducks) was transferred into group 1 (inoculated ducks) at 1 dpi. To determine virus transmission during the period after acute infection (hereafter referred to as the post-acute infection stage) groups 3 (inoculated) and 4 (contact) were mixed at 10 dpi. To avoid the transmission from the contaminated room environment, the ten inoculated ducks of group 3 were securely transferred into the uncontaminated room containing group 4. Ducks in all groups were monitored after the virus inoculation or after they were mixed. Oral and cloacal swabs were collected once before the inoculation date and daily after the inoculation date; all the swab samples were stored at −80°C before testing. Sera were collected from each duck as 2–3 ml clotted blood from the wing vein with the following schedule: once before inoculation for all groups; at 8 and 15 dpi for groups 1 and 2 (equivalent to 7 and 14 days post-contact (dpc) for group 2); at 8, 15, 22, 29, and 34 dpi for group 3; and 22, 29 and 34 dpi (12, 19, 24 dpc) for group 4. For welfare reasons, clinically affected ducks were euthanized with intravenous pentobarbital sodium (Lethabarb, Virbac Animal Health, Australia) once they developed moderate clinical signs.

### Serology

The hemagglutination inhibition (HI), virus neutralization (VN) and blocking enzyme- linked immunosorbent assay (bELISA) tests were used to detect the presence of antibodies prior to and after virus inoculation. Blood samples in EDTA were collected from each duck at the designated times. After overnight incubation at 4°C, blood was centrifuged at 1000 g for 10 minutes to separate the sera. Sera then were heat-treated at 56°C for 1 hour immediately after separation. Homologous antigen, either inactivated or live homologous virus (IDN34368), was used in the HI and the VN tests respectively, while recombinant influenza nucleoprotein (NP) antigen was used for bELISA. For HI and VN tests, sera were first diluted 1∶2 before testing; sera for HI were diluted (adsorbed) with 10% chicken red blood cells (RBCs) or with 10% horse RBCs, while for VN sera were diluted with phosphate buffer saline (PBS). These sera were further diluted 1∶2 with PBS at the start of the HI and VN tests; therefore, the minimal detectable titre given by the HI and VN tests was 4. For bELISA, 1∶10 diluted sera in serum diluent (PBS with 0.05% Tween 20 and 1% skim milk) were used and the cut-off was set at 60% inhibition. All the tests were conducted using methods as previously described [31].

### Virology

Virus isolation in eggs was performed for all oral and cloacal swab media collected from ducks in groups 1 and 2. For group 3, it was conducted on the swab media obtained from ducks at 1–15 dpi and from selected days afterward (17, 20, 24, and 29 dpi), whereas for group 4, it was performed for the swab media of ducks from 1, 5 and 7 dpc. For this, 0.2 ml undiluted swab medium was inoculated into the allantoic cavity of 9 to 11-day-old embryonated eggs (3 eggs inoculated per sample). Eggs were observed for the death of embryos each day for 3 days after inoculation. The allantoic fluids from all dead eggs were tested for the presence of influenza virus by the HA test using standard methods [32]. A swab was considered virus-positive if at least one of the inoculated eggs was HA-positive.

For three ducks that showed clinical signs during the experiment (ducks #85, #94, #97), 100–150 mg of brain, heart, lung, pharynx, trachea, air sacs, spleen, pancreas, small intestine and skeletal muscle were collected following euthanasia into tubes containing 1 ml of transport media and 0.4 to 0.5 µg of 1.0 mm silicon carbide beads (Daintree Scientific, St. Helens, Tasmania, Australia). Tissue homogenates were titred for virus in Vero cells grown in culture media (EMEM plus HEPES, glutamine, penicillin and streptomycin, fungizone, and 10% fetal calf serum/FCS), as described previously [28]. Because the dilutions started from 1∶10, the lowest detectable titres, equivalent to CPE occurring in a single well at the lowest dilution, was 10^0.7^ 50% tissue culture infectious doses (TCID_50_) per 0.1 ml. The TCID_50_ was calculated by the Reed and Muench method [33].

### RNA extraction and real-time reverse transcriptase-PCR (rRT-PCR)

**Figure 1:**
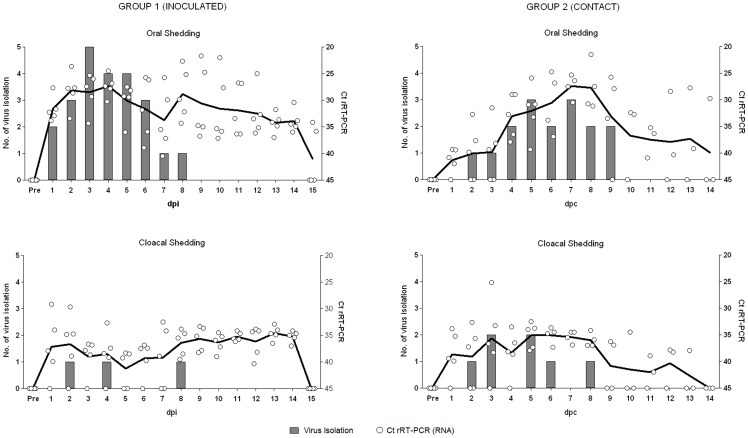
Virus isolation and H5 RNA Ct values for oral and cloacal swabs from the inoculated ducks (group 1) and the acute stage-contact ducks (group 2). Five contact ducks of group 2 (charts at right) were mixed with five inoculated ducks of group 1 (charts at left) at one day post inoculation (1 dpi). Swabs were obtained from all ducks in group 1 for 1–15 dpi, while those for group 2 were collected from 5 ducks from 1–5 day post-contact (dpc), 4 ducks from 6–9 dpc and 3 ducks from 10–15 dpc as 2 ducks were euthanized at 5 and 9 dpc. Vertical column bars indicate the number of ducks positive for virus isolation in embryonated chicken eggs, while white circles indicate the H5 RNA Ct values from rRT-PCR. Circles for Ct values have been staggered to avoid overlap. The means of Ct values from each time point are connected (solid black line); undetectable Ct values were given the value of 45 for calculation of the means.

**Figure 2:**
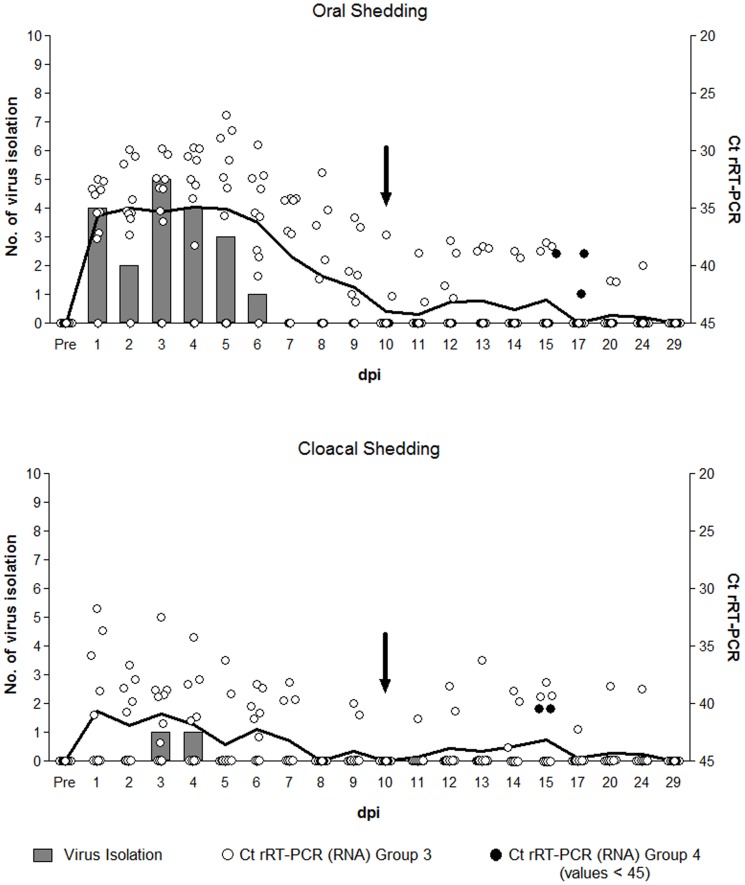
Virus isolation and H5 RNA Ct values for oral and cloacal swabs from the inoculated ducks (group 3) and the post-acute stage contact ducks (group 4). Swabs from ducks of group 3 (n = 10) were collected 1–15 day post inoculation (dpi) and at 17, 20, 24 and 29 dpi. Nine contact ducks (group 4) were mixed with the inoculated ducks of group 3 at 10 dpi (arrow). Virus isolation and rRT-PCR for group 4 was performed on oral and cloacal swabs collected from all the contact ducks at 1, 5 and 7 days post contact (dpc) (equivalent to 11, 15, 17 dpi); none of these swabs were virus isolation positive and only five swabs from different contact ducks had detectable Ct values (black circles). The mean Ct values for group 3 from each time point are connected (solid black line); undetectable Ct values were given the value of 45 for calculation of the means.

RNA extraction and rRT-PCR were performed from the same oral and cloacal swab media as used for the virus isolation. Viral RNA was extracted from 50 µl of each swab medium. Viral RNA was also extracted from tissue homogenates of the two contact ducks from group 2, which were euthanized at 6 and 10 dpi. The MagMAX™-96 Viral RNA Isolation Kit (Ambion®, Applied Biosystem) with the Ambion® 96-well Magnetic-Ring stand were used for the RNA extraction as per the manufacturer's protocol. RNA was then tested for the presence of hemagglutinin (HA) gene of H5 subtype using a multiplex TaqMan assay with AgPath-ID One-Step RT-PCR Kit (Applied Biosystem). The assay consists of two sets of primers and probes targeting two different regions (C-terminus and N-terminus) of the hemagglutinin gene (sequences available upon request). The rRT-PCR assays were conducted using AAHL's standard conditions: 45°C for 10 min, 95°C 10 min, followed by 45 cycles of 95°C 15 sec and 60°C 45 sec. In this study, swab media with an undetectable Ct were assigned a value of 45 for calculation of the mean Ct scores for each group and for presentation of data (Figure 1 and 2).

### Histopathology and immunohistochemistry

A range of tissues and structures, including heart, brain, spleen, liver, kidney, lung, trachea, air sacs, esophagus, proventiculus, gizzard, thymus, bursa, pancreas, small intestines, cecal tonsil, third eyelid and head samples, were obtained from three euthanized ducks which showed clinical signs of disease during the experiment (ducks #85, #94, #97). Tissues were placed into 10% neutral buffered formalin and fixed for 1–7 days, before they were processed for histology and immunohistochemistry (IHC) staining for detection of tissue lesions and influenza A nucleoprotein antigen, respectively. Procedures used to perform the histology and IHC followed the method as previously described [30].

## Results

In this study, the trends of infectious virus and viral RNA shedding of ducks inoculated with Indonesian clade 2.1 H5N1 HPAI virus were determined by virus isolation and the monitoring of contact ducks and by rRT-PCR of oral and cloacal swabs, respectively. Prior to challenge, all the ducks were antibody negative by HI, VN and bELISA tests, swabs were all negative by virus isolation in embryonated eggs and H5 rRT-PCR Ct values were undetectable (≥45). These test results indicate that the ducks had not had prior exposure to influenza virus.

### Serology

The antibody responses were measured by bELISA, HI using chicken RBCs (HI-C), HI using horse RBCs (HI-H), and VN tests and these responses were used to confirm that infection had occurred in individual ducks.

The antibody response in inoculated (group 1) and acute stage contact ducks (group 2) is shown in Table 1. At 8 dpi, 100% (5/5) of the inoculated ducks had seroconverted, as detectable antibodies shown by bELISA (>60% inhibition) and both HI tests, but no neutralizing antibodies were detected. At the same time (equivalent to 7 dpc), none of the contact ducks of group 2 had seroconverted by the HI and VN tests, but 60% (3/5, including duck #94 at 5 dpc) were positive by bELISA. The levels of detectable HI and VN antibodies increased about 1–3 log_2_ from 8 to 15 dpi, whereas the levels of bELISA appeared to remain steady over the same time. At 9 dpc, contact duck #85 had seroconverted by the bELISA and both HI tests, but not the VN test. At termination (15 dpi or 14 dpc, respectively), all 5 inoculated ducks and 2 of the 3 remaining contact ducks had significant antibodies by all tests. One contact duck (#71) developed only a marginal bELISA signal at 14 dpc, despite indication of infection since 2 dpc.

**Table 1:** Serum antibody titers and viral detection in the H5N1 HPAI virus-inoculated (group 1) and acute stage contact (group 2) ducks.

	Pre-Inoculation				8 dpi				15 dpi					
Bird ID	ELISA	HI-C	HI-H	VN	ELI SA	HI-C	HI-H	VN	ELI SA	HI-C	HI-H	VN	VI^a^	rRT-PCR^b^
**Group 1**														
#73	36	<4	<4	<4	107	16	32	<4	104	128	128	128	+	27.8
#75	26	<4	<4	<4	103	8	32	<4	103	64	128	128	+	23.6
#77	34	<4	<4	<4	99	16	64	<4	105	128	128	128	+	26.8
#83	36	<4	<4	<4	101	32	64	<4	99	256	512	128	+	21.6
#92	30	<4	<4	<4	104	16	16	<4	104	128	256	128	+	24.5
**Group 2**														
#71	27	<4	<4	<4	55	<4	<4	<4	63	<4	<4	<4	+	30.5
#84	17	<4	<4	<4	54	<4	<4	<4	104	128	256	16	+	21.5
#85^c^	37	<4	<4	<4	102	<4	<4	<4	93	4	16	<4	+	24.7
#87	33	<4	<4	<4	68	<4	<4	<4	90	64	128	32	+	26.4
#94^c^	26	<4	<4	<4	64	<4	<4	<4	nd	nd	nd	nd	+	30.9

Five ducks inoculated with H5N1 HPAI virus (group 1) and five naive contact ducks (group 2) were mixed at one day post inoculation (dpi). Sera were collected at 4 days pre-inoculation for both groups and at 8 and 15 dpi (equivalent to 7 and 14 days post contact (dpc) respectively for group 2). Sera were tested with blocking ELISA (values are % inhibition; cut-off = 60%), HI using chicken RBCs (HI-C) and horse RBCs (HI-H), and virus neutralization (VN) tests. nd: not done.

^a^ Summary of virus isolation (VI): a+symbol indicates that virus was isolated at least once from either oral or cloacal swab from the duck during the duration of the trial.

^b^ Summary of Ct values for real-time reverse transcription polymerase chain reaction (rRT-PCR) results: the value is the lowest detected for the duck from either oral or cloacal swabs during the duration of the trial.

^c^ Ducks 94 and 85 developed disease during the experiment; therefore the last sera were obtained when they were euthanized, for welfare reasons, at 5 dpc (shown under 8 dpi) and 9 dpc (shown under 15 dpi), respectively.

In group 3 (inoculated), the proportion of ducks that had seroconverted at 8 dpi varied among the different tests: 9/10 (bELISA), 8/10 (HI-H), 7/10 (HI-C) and 5/10 (VN) (Table 2). At the later sampling times (15, 22, 29, 34 dpi), these proportions were more comparable, with all ducks, except one, having seroconverted by all four tests. Duck #80 remained seronegative by bELISA and without detectable HI and VN antibodies throughout the experiment. In contrast, influenza antibodies were not detected to the end of the study at 34 dpi (24 dpc) in any of the nine post-acute stage contact ducks in group 4 after they were mixed with the inoculated ducks of group 3.

**Table 2:** Serum antibodies and viral detection in the H5N1-infected (group 3) and post acute stage contact (group 4) ducks.

Bird	Pre-Inoculation				8 dpi				15 dpi				22 dpi				29 dpi				34 dpi				VI^a^	rRT-PCR^b^
ID	ELI SA	HI-C	HI-H	VN	ELI SA	HI-C	HI-H	VN	ELI SA	HI-C	HI-H	VN	ELI SA	HI-C	HI-H	VN	ELI SA	HI-C	HI-H	VN	ELI SA	HI-C	HI-H	VN		
**Group 3**																										
#72	29	<4	<4	<4	104	64	128	16	104	64	64	64	106	256	128	1024	108	128	128	512	97	64	64	1024	+	29.7
#76	35	<4	<4	<4	103	8	32	4	103	64	128	32	106	128	64	256	107	128	128	256	102	64	128	512	+	26.9
#80	28	<4	<4	<4	25	<4	<4	<4	24	<4	<4	<4	35	<4	<4	<4	30	<4	<4	<4	38	<4	<4	<4	−	38.0
#81	50	<4	<4	<4	95	64	64	8	104	256	256	128	102	256	128	1024	108	256	256	512	99	128	64	256	+	28.2
#82	23	<4	<4	<4	88	<4	<4	<4	88	8	8	8	96	8	8	16	84	8	8	8	77	4	8	16	+	33.8
#90	39	<4	<4	<4	88	4	8	<4	90	32	16	32	86	16	8	8	80	8	4	16	68	8	4	32	−	35.9
#93	31	<4	<4	<4	100	32	32	8	104	64	64	128	107	128	64	512	107	64	32	256	101	64	64	256	+	30.5
#97	33	<4	<4	<4	105	64	128	32	104	256	128	256	107	256	128	256	106	128	128	512	100	128	128	512	+	29.9
#98	36	<4	<4	<4	94	<4	32	<4	94	32	32	32	106	64	32	128	107	64	64	128	102	64	32	256	+	33.2
#99	18	<4	<4	<4	85	4	32	<4	101	64	64	128	105	128	32	512	105	128	64	512	104	128	64	512	+	28.9
**Group 4**																										
#74	36	<4	<4	<4	nd	nd	nd	nd	nd	nd	nd	nd	30	<4	<4	<4	22	<4	<4	<4	16	<4	<4	<4	−	38.9
#78	35	<4	<4	<4	nd	nd	nd	nd	nd	nd	nd	nd	31	<4	<4	<4	32	<4	<4	<4	18	<4	<4	<4	−	-
#79	22	<4	<4	<4	nd	nd	nd	nd	nd	nd	nd	nd	34	<4	<4	<4	25	<4	<4	<4	33	<4	<4	<4	−	40.8
#86	30	<4	<4	<4	nd	nd	nd	nd	nd	nd	nd	nd	27	<4	<4	<4	18	<4	<4	<4	34	<4	<4	<4	−	-
#89	28	<4	<4	<4	nd	nd	nd	nd	nd	nd	nd	nd	33	<4	<4	<4	30	<4	<4	<4	19	<4	<4	<4	−	40.7
#91	27	<4	<4	<4	nd	nd	nd	nd	nd	nd	nd	nd	27	<4	<4	<4	36	<4	<4	<4	24	<4	<4	<4	−	38.9
#95	33	<4	<4	<4	nd	nd	nd	nd	nd	nd	nd	nd	8	<4	<4	<4	26	<4	<4	<4	18	<4	<4	<4	−	-
#96	17	<4	<4	<4	nd	nd	nd	nd	nd	nd	nd	nd	9	<4	<4	<4	29	<4	<4	<4	26	<4	<4	<4	−	-
#100	18	<4	<4	<4	nd	nd	nd	nd	nd	nd	nd	nd	30	<4	<4	<4	23	<4	<4	<4	25	<4	<4	<4	−	42.4

Ten ducks (group 3) inoculated with H5N1 HPAI virus were mixed with nine naive contact ducks (group 4) at 10 day post inoculation (dpi). Sera were collected at 4 days pre-inoculation for both groups and at 8, 15, 22, 29, and 34 dpi for both groups, which corresponded to 12, 19 and 24 days post contact (dpc) for group 4. Sera were tested using blocking ELISA (values are % inhibition; cut-off = 60%), HI with chicken RBCs (HI-C) and horse RBCs (HI-H), and virus neutralization (VN) tests. nd: not done.

^a^ Summary of virus isolation (VI): a+symbol indicates that virus was isolated at least once from an oral or cloacal swab from the duck during the duration of the trial.

^b^ Summary of Ct values for real-time reverse transcription polymerase chain reaction (rRT-PCR) results: the value is the lowest detected for the duck from either oral or cloacal swabs during the duration of the trial.

### Viral shedding

The patterns of viral shedding, including route and duration, were determined by detecting infectious virus or H5-RNA in oral and cloacal swabs of ducks from groups 1, 2 and 3. Because none of the contact ducks in group 4 seroconverted, oral and cloacal swabs sampled only from selected days (1, 5 and 7 dpc) were tested for virus isolation and rRT-PCR in order to support the evidence of lack of viral infection.

#### Groups 1 and 2: acute infection stage shedding and transmission

Virus isolation and H5 RNA Ct values from oral and cloacal swabs of ducks in groups 1 and 2 are shown in Figure 1. The means of Ct values were incorporated to demonstrate the kinetics of RNA shedding. On at least one occasion in the study, all the ducks in both groups were virus isolation positive and had rRT-PCR Ct values ≤30.9 (Table 1, virology results), confirming that all ducks in these groups became infected.

In group 1, Ct values <40 were detected in oral swabs from all the five ducks at 1 dpi and this was consistently found at the later sampling times (Figure 1 and Table S1). At 1–7 dpi the proportion of oral swabs with detectable H5-RNA was always higher than that of cloacal swabs, but at 8–14 dpi the relative proportions in oral and cloacal swabs were more similar. The relative quantity of RNA shedding from oral swabs was higher (Ct values: 27.3–34.9) than cloacal swabs (Ct values: 34.5–38.7). Infectious virus was isolated in oral swabs from 2 of 5 ducks at 1 dpi, but all cloacal swabs were virus isolation negative at this time. The peak of infectious virus and viral RNA shedding was seen around 3 and 4 dpi, with a high proportion of ducks being virus isolation positive and a high concentration of viral RNA found on those days, especially in the oral swabs. In agreement with the results of viral RNA shedding, the proportion of virus isolation positive oral swabs was consistently higher than that of cloacal swabs. Although H5-RNA was detected in both cloacal and oral swabs until 14 and 15 dpi, respectively, infectious virus was only detected up to 8 dpi.

In group 2, H5-RNA was detected in both oral and cloacal swabs of the contact ducks, starting at 1 dpc (Figure 1, Table S1). The relative amount of oral-RNA shedding (Ct values: 26.2–37.5) was always higher than cloacal-RNA shedding (Ct values: 33.4–40.5), with the exceptions at 1 to 3 dpc. Oral-RNA shedding peaked at 7 dpc and in one bird continued to be shed through the oral route until the end of the study at 14 dpc. Infectious virus was detected in cloacal and oral swabs from 2 dpc to 8 or 9 dpc, with the daily proportion of virus isolation positive cloacal swabs often lower than oral swabs (Figure 1). Infectious virus was detected at least once in each bird. The peak of infectious virus shedding occurred around 5 and 7 dpc.

#### Groups 3 and 4 – post-acute infection stage shedding and transmission

In group 3, the proportion of oral swabs that contained detectable H5-RNA was consistently higher than with cloacal swabs (Figure 2, Table S2). The mean Ct value was also higher in oral swabs than in cloacal swabs over this period. H5-RNA was detected daily in oral swabs of four of 10 ducks from 1 to 9 or 10 dpi, but only intermittently detected afterwards. On the other hand, in cloacal swabs H5-RNA was intermittent throughout most of the study period (Table S2). At 1 dpi, infectious virus was detected in oral swabs from 4 of 9 RNA-positive ducks and it was detected from the same swab type up to 6 dpi (Figure 2). In contrast, virus isolation positive cloacal swabs were found only at 3 and 4 dpi, from the same duck. Although one duck (#80) in this group was H5-antibody negative for 34 days observation, low H5 RNA levels (Ct value: 38.0–42.6) were detected intermittently in its oral (at 8, 10 and 15 dpi) and cloacal swabs (at 6 and 12 dpi), but no viable virus was detected.

In post-acute stage contact ducks (Group 4), infectious virus was not found in any oral or cloacal swabs at the selected days: 1, 5 and 7 dpc (Figure 2, Table 2). However, low levels of H5-RNA (Ct values ≥38.9) were detected in swab media from 5 of the 9 ducks in this group: 3 oral and 2 cloacal swabs at 5 and 7 dpc (Figure 2).

### Outcomes of H5N1 virus infection

There were no clinical signs in the majority of ducks in all groups after virus inoculation or contact, indicating that infection by the H5N1 HPAI virus used in this study was largely asymptomatic in ducks. However, three ducks developed signs of disease. Duck #97 in Group 3 developed mild head tilt which persisted until the termination of the trial at 34 dpi. Histological observation on this duck found mild chronic focal lymphocytic inflammation of the myocardium, Eustachian tube and a ganglion of the inner ear. The significance of these lesions is not clear. No viral antigen was detected in any of the tissues from this duck.

Two of five acute infection stage contact ducks of group 2 (duck #94 and #85) showed recumbency and depression for 1–2 days before they were euthanized at 5 and 9 dpc respectively. On necropsy examination a white membrane over the peritoneal organs and airsacs, and excessive peritoneal fluid, were noted in both ducks.

**Figure 3:**
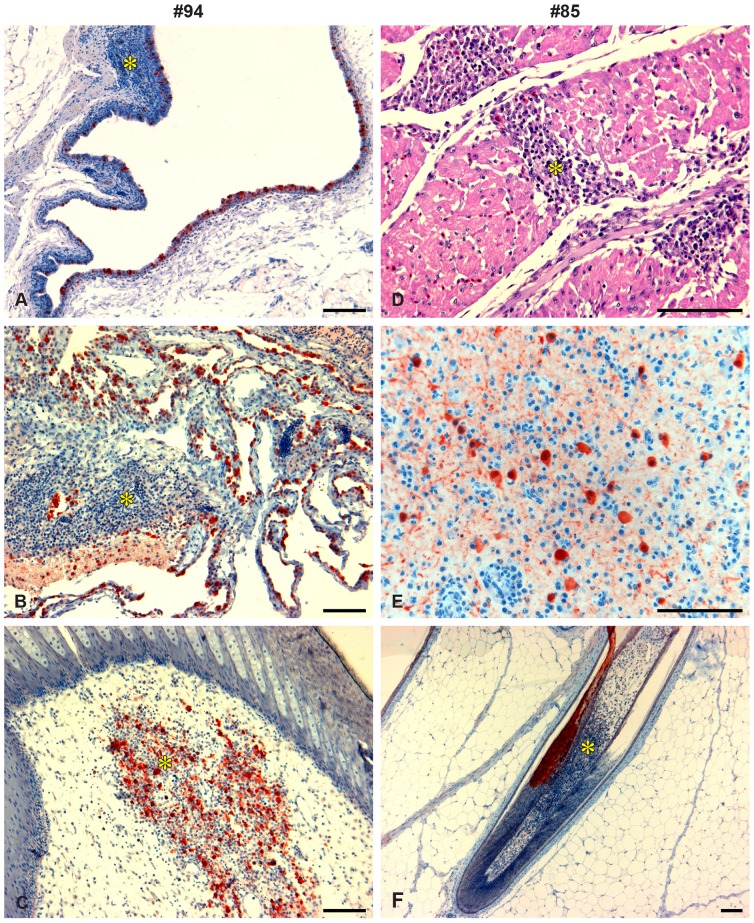
Histopathology and immunohistochemistry of H5N1 HPAI virus infection in acute stage contact-infected ducks #94 and #85. Ducks #94 (left, A–C) and #85 (right, D–F) were euthanized at 5 and 9 days post contact (dpc) following disease signs. A. Infraorbital sinus, showing influenza viral nucleoprotein antigen in epithelium and mononuclear cell inflammation (*). B. Air sac, showing abundant viral antigen in the epithelial membranes and in fibrinous exudates, and mononuclear cell infiltration (*); C. Feather, with abundant antigen staining associated with mixed cell inflammation (*) in the pulp; D. Heart, showing severe, sub-acute, diffuse mononuclear cell myocarditis (*); no antigen was detected in myocardium E. Brain, showing antigen in neuronal tissue associated with mononuclear cell encephalitis; F. Feather, with mononuclear cell pterilitis (*) observed in the pulp, and antigen staining in the epidermis. Haematoxylin and eosin stain (Fig. D) and immunohistochemistry stain (brown color) for influenza A virus nucleoprotein (Fig. A, B, C, E, F). All scale bars = 100 µm.

Histological examination of tissues of duck #94 found that disease consisted of inflammation of the respiratory system with systemic viral spread. There was severe acute localized necrotizing mononuclear cell airsacculitis, mild edema in the lungs and mononuclear cell inflammation in the pulp of occasional feathers. Abundant antigen was present in the epithelial tissues of the respiratory tract, and small to moderate amounts of antigen were found in a range of other tissues (Figure 3A, 3B and 3C; Table 3). In this duck, the tissues with the highest titers of virus and highest viral RNA levels were air sac, trachea and spleen (Table 3).

**Table 3:** Virus distribution in tissues of the two clinically affected and euthanized acute stage-contact ducks in group 2, as determined by immunohistochemistry, virus isolation and rRT-PCR.

	Duck #94	Duck #85
	(5 dpc)	(9 dpc)
**Immunohistochemistry**	**Antigen score** a	
*Epithelia of respiratory tract:*		
Infraorbital sinus	+++	−
Trachea	+	−
Bronchi	++	−
Lung air capillaries	+	−
Air sacs	+++	+
*Brain* b	−	++
*Lymphoid tissue:*		
Bursa (medulla)	+	−
Thymus (medulla)	+	−
Conjunctiva	+	−
*Connective tissue, integument and associated tissues of head:*		
Endosteum	+	+
Periosteum	+	+
Feather epidermis	−	+++
Feather pulp	+	−
*Muscle:*		
Myocardium	+	−
Pectoral skeletal muscle	−	+
**Virus titration in Vero cells and rRT-PCR**		
	**Virus titre** c **(Ct value)**	
Brain	<0.7 (31.4)	<0.7(19.6)
Heart	3.7 (20.7)	<0.7 (23.6)
Spleen	4.7 (19.2)	<0.7 (33.4)
Lung	2.7 (23.2)	<0.7 (30.1)
Pharynx	3.5 (23.9)	<0.7 (27.3)
Trachea	5.2 (19.9)	0.7 (27.8)
Air sacs	5.7 (15.5)	<0.7 (28.6)
Pancreas	3.7 (25.6)	<0.7 (32.2)
Small intestine	2.5 (27.0)	<0.7 (32.9)
Skeletal muscle	1.2 (26.3)	<0.7 (32.3)

^a^ Influenza nucleoprotein antigen staining was present in single cells or small foci (+) or in localized clusters with moderate (++) to abundant (+++) quantities.

^b^ Antigen found in neural tissues including neuron cell bodies, neuroglia and neuropil.

^c^ Log_10_ TCID_50_/0.1 ml of tissue homogenate.

In duck #85, disease was characterized by generalized severe sub-acute inflammation of multiple organ systems, including heart, brain and airsacs. Lesions consisted of severe sub-acute diffuse myocarditis (Figure 3D), severe localized (cerebral) mononuclear cell encephalitis, severe sub-acute diffuse airsacculitis, moderate sub-acute diffuse mononuclear cell pectoral myositis, and mild mononuclear cell pterylitis in the pulp of occasional feathers. Small to moderate (although locally dense) amounts of antigen were found in the brain and other tissues (Figure 3; Table 3). In this duck, the greatest abundance of antigen was found in the feathers. Virus could not be isolated from any tissues except for trachea, which yielded a very low titre of virus. However, viral RNA was detected in all tissues tested, with the brain showing a relatively high viral RNA load (Ct = 19.9) (Table 3). It is probable that the peak of viral infection was passed at the time of euthanasia, as the levels of antigen and viral loads were relatively low despite severe histological lesions.

## Discussion

In this study, we investigated the shedding of an Indonesian clade 2.1 H5N1 HPAI virus by domestic ducks after the acute infection stage, in an attempt to assess the potential impact of long-term infection of ducks in the evolution of H5N1 HPAI viruses in Indonesia. Although we found that ducks will shed and efficiently transmit H5N1 HPAI virus during acute infection, we found no evidence of shedding of viable virus or transmission after 10 dpi. None of the contact ducks (group 4) that were mixed with the inoculated ducks (group 3) after 10 dpi seroconverted or shed infectious virus. In contrast, in a separate contact group that was mixed during the acute stage of infection (at 1 dpi) virus was readily transmitted from infected ducks, which shed significant amounts of infectious virus and viral RNA. Although continued shedding of H5 viral RNA was shown in the inoculated ducks (group 3) up to 24 dpi, the negative virus isolation results from swabs and lack of evidence of infection in the post-acute infection stage contact ducks (group 4) indicated the absence of infectious virus shedding after 10 dpi. None of these contact ducks seroconverted although H5 RNA was detected in oral or cloacal swabs of a small number of ducks in group 4. It is likely that virus could not persist in the primary inoculated hosts because of the development of antibodies against H5N1 HPAI virus in those ducks by day 8 post-inoculation.

A previous study by Hulse-Post *et al.* [21] found that prolonged shedding of infectious H5N1 HPAI virus occurred in mallard ducks for up to 17 dpi despite seroconversion to the challenge virus. They hypothesize that neutralizing antibody does not completely eliminate all variants of the viral population, allowing repetitive infections of the host with the surviving variants. The extended, sub-clinical, infection period allows the host to transmit the immunologically novel variants to other hosts. However, our data indicate that, in domestic ducks infected with Indonesian H5N1 HPAI virus, the infectious period is short and its termination coincides with the rise of antibody. While our data do not preclude that viruses generated at the end of the infectious period may be antigenically different due to immunological selection, they indicate that the potential for this mechanism is considerably less than indicated by Hulse-Post *et al.* [21]. Given the short duration of the infectious period, the evolution of completely new immunological variants arising within a single duck host infected with this clade 2.1 virus, would appear to be an unlikely outcome.

Ducks were used as contacts in order to add an extra level of sensitivity for virus detection. Detection of transmission to a live host system would increase the probability of detection of viral shedding over the use of only *in vitro* methods such as swabbing. The use of ducks and not a different species, such as chickens, was considered appropriate as intra-species transmission is probably more efficient. Previous work at AAHL with H5N1 HPAI viruses indicated that Pekin ducks are a more sensitive recipient species within contact-transmission trials than are chickens (J. Bingham, unpublished data). In this study, relatively large group sizes (10 and 9) of ducks were used to optimize the probability of transmission, given that shedding was expected to be low during the post-acute stage.

The results of this trial indicate that viral RNA is highly persistent. In both inoculated groups (groups 1 and 3) and in the acute infection stage-contact group (group 2), shedding of viral RNA was detected over longer periods than shedding of infectious virus. In the inoculated infected ducks (group 3), high level RNA shedding was only detected until 8–9 dpi. Viral RNA was shed intermittently at low levels at the later days of the trial in these ducks and in the post-acute infection stage of contact ducks (group 4), which otherwise showed no evidence of infection.

The rRT-PCR method is quick, safe and can handle larger numbers of samples more easily than virus isolation for H5N1 HPAI virus screening. At AAHL, the cycle threshold (Ct) for diagnostic positivity is normally set at <40, based on previously determined assays on positive control H5 RNA. However our data indicate that the stage of infection cannot easily be determined from rRT-PCR data alone, as H5 RNA continues to be detected weeks after infectious virus has disappeared. In addition, in the context of infectious risk, diagnosis of HPAI virus in ducks by detection of RNA is less meaningful than the level and duration of infectious virus shedding. The finding of Ct values below the threshold of positivity in ducks that otherwise have no evidence of infection indicates that contact with infectious birds may induce false positives by rRT-PCR. Although this molecular assay is an appropriate method for surveillance, its data must be interpreted carefully when making decisions on the infection status of individual birds, given that intra-flock cross contamination may occur during and shortly after an outbreak.

Consistent with some experimental studies using other Eurasian lineage H5N1 HPAI viruses [10], [27]–[29], the present study demonstrated that the viral shedding in ducks, either infectious virus or H5 RNA, was more pronounced by the oral route than by the cloacal route. Our previous studies [28], [30] indicated that the respiratory tract of ducks is the primary site for virus replication and the main source from which virus is shed from the oral cavity. This is contrary to LPAI viruses in which high viral concentration was found in fecal matter of ducks [10], [34] and that LPAI virus transmission relies on fecal to oral transmission [35], [36]. It would appear that with H5N1 HPAI, virus transmission among ducks is either through the air-borne route, where virus is expelled from the respiratory tract in droplets, or through dabbling, a behavioral feature of mallard-type ducks, which would facilitate the flushing of oral virus into water.

The inoculation of H5N1 HPAI virus into ducks is quickly followed within one to two days by oral and cloacal shedding; and the virus is efficiently transmitted at this time into naïve contact ducks. Interestingly, not all inoculated ducks developed evidence of infection: one duck (#80) did not seroconvert, no infectious virus was detected from it and Ct values for H5 RNA were intermittent and remained above 38.0. This duck appeared to have resisted not only the inoculation but also the contact challenge from its infected flockmates. Two other ducks (#82 and #90) seroconverted with low antibody titers, indicating infection had occurred, although only one of the ducks was positive on virus isolation. These data indicate that infection is variable at the dose administered in this study, even when followed by exposure to infected ducks.

In contrast with the inoculated ducks, of which only one of 15 showed mild neurological signs, two of five acute stage contact ducks developed clinical signs severe enough to warrant euthanasia. This suggests that contact-transmission may be more likely to induce severe disease or that more pathogenic mutants arose during the course of the study. Further investigation is important to understand the effects of transmission routes and to identify whether viruses isolated from these ducks harbor genetic mutations related to pathogenicity.

In conclusion, this study shows that domestic ducks efficiently acquired infection of Indonesian H5N1 HPAI virus through transmission during the acute infection stage. However, viral shedding occurred during a relatively short period and was eliminated coincident with the rise of influenza antibodies. Our studies indicated that there is no evidence of persistent shedding of infectious Indonesian H5N1 HPAI clade 2.1 virus in experimentally infected domestic ducks; they suggest that long-term viral infection and shedding in ducks is unlikely to be a significant factor in the ecological cycle and persistence of H5N1 HPAI virus. This supports the view that antigenic drift, which allows evolved viral types to re-infect a population of hosts, would take place over several transmission cycles in naive hosts, rather than in single hosts with extended infections. Models for assessing risk and for viral maintenance in duck populations should take this information in account.

## Supporting Information

Table S1 Viral isolation and Ct rRT-PCR values for oral and cloacal swabs of the H5N1-inoculated (group 1) and acute infection stage-contact (group 2) ducks.
(DOCX)

Table S2 Viral isolation and Ct rRT-PCR values for oral and cloacal swabs of the H5N1-inoculated ducks (group 3).
(DOCX)
