# Supplementary material for: Experimentally Infected Domestic Ducks Show Efficient Transmission of Indonesian H5N1 Highly Pathogenic Avian Influenza Virus, but Lack Persistent Viral Shedding
Source: PLoS One. 2014 Jan 2;9(1):e83417. doi: 10.1371/journal.pone.0083417 (PMC3879242; doi:10.1371/journal.pone.0083417)
Supplement: Table S1 — Viral isolation and Ct rRT-PCR values for oral and cloacal swabs of the H5N1-inoculated (group 1) and acute infection stage-contact (group 2) ducks. (DOCX) [file pone.0083417.s001.docx]

**Table S1.** Viral isolation and Ct rRT-PCR values for oral and cloacal swabs of the H5N1-inoculated (group 1) and acute infection stage-contact (group 2) ducks.

| **Group 1 (inoculated ducks)** | | | | | | | | | | | |
| --- | --- | --- | --- | --- | --- | --- | --- | --- | --- | --- | --- |
|  | **Oral Swab** | | | | |  | **Cloacal Swab** | | | | |
| **Bird ID** | **#73** | **#75** | **#77** | **#83** | **#92** |  | **#73** | **#75** | **#77** | **#83** | **#92** |
| Pre-Inoculation | - | - | - | - | - |  | - | - | - | - | - |
| 1 dpi | 33.8 | 32.9 | 31.6 | ***27.6*** | ***32.2*** |  | - | 37.9 | 34.0 | 29.1 | 39.9 |
| 2 dpi | ***33.4*** | ***23.6*** | 27.6 | 27.7 | ***28.6*** |  | - | 38.9 | 34.8 | ***29.6*** | 34.7 |
| 3 dpi | ***34.3*** | ***25.3*** | ***29.3*** | ***26.0*** | ***27.4*** |  | 36.8 | - | 37.8 | 38.6 | 36.6 |
| 4 dpi | ***27.8*** | ***26.8*** | 30.2 | ***27.3*** | ***24.5*** |  | ***32.6*** | 37.4 | 39.1 | - | 38.4 |
| 5 dpi | ***28.1*** | ***29.4*** | 36.0 | ***29.2*** | ***27.5*** |  | 38.3 | 38.5 | - | - | 39.2 |
| 6 dpi | ***35.9*** | 31.8 | 38.9 | ***25.7*** | ***26.1*** |  | - | 36.8 | 37.5 | 39.7 | 37.4 |
| 7 dpi | 40.5 | 37.1 | 35.5 | ***25.7*** | 29.9 |  | 38.9 | 32.5 | - | - | 34.1 |
| 8 dpi | 32.1 | 29.8 | 34.3 | ***22.6*** | 25.1 |  | 35.5 | 38.5 | 39.5 | ***33.8*** | 34.6 |
| 9 dpi | 34.7 | 36.7 | 35.0 | 21.6 | 24.7 |  | 38.2 | 37.8 | 33.3 | 35.1 | 33.6 |
| 10 dpi | 35.3 | 37.1 | 35.8 | 22.0 | 27.6 |  | 34.5 | 37.1 | 35.9 | 35.2 | 39.0 |
| 11 dpi | 33.4 | 36.3 | 26.8 | 36.3 | 26.7 |  | 36.1 | 34.1 | 34.6 | 35.6 | 35.7 |
| 12 dpi | 35.1 | 36.1 | 33.5 | 25.0 | 32.6 |  | 33.8 | 34.3 | 34.1 | 40.3 | 38.1 |
| 13 dpi | 33.6 | 34.1 | 34.8 | 37.0 | 31.7 |  | 34.0 | 34.9 | 34.6 | 32.9 | 36.5 |
| 14 dpi | 34.9 | 34.5 | 33.8 | 36.0 | 30.4 |  | 34.1 | 35.0 | 34.6 | 35.4 | 37.0 |
| 15 dpi | - | - | - | 34.1 | 35.8 |  | - | - | - | - | - |
| **Group 2 (acute infection stage-contact ducks)** | | | | | | | | | | | |
|  | **Oral Swab** | | | | |  | **Cloacal Swab** | | | | |
| **Bird ID** | **#71** | **#84** | **#85** | **#87** | **#94** |  | **#71** | **#84** | **#85** | **#87** | **#94** |
| Pre-Inoculation | - | - | - | - | - |  | - | - | - | - | - |
| 1 dpc | 42.0 | - | 39.3 | 40.8 | 39.4 |  | - | 39.4 | 35.2 | 33.8 | 39.9 |
| 2 dpc | ***32.7*** | - | - | 37.6 | 39.8 |  | - | - | 37.2 | 35.6 | ***32.6*** |
| 3 dpc | - | 39.3 | - | 38.1 | ***31.5*** |  | 38.3 | 36.6 | ***25.1*** | ***33.2*** | - |
| 4 dpc | 37.9 | 36.5 | 29.0 | ***29.0*** | ***32.7*** |  | 38.6 | - | 33.5 | 36.5 | 38.1 |
| 5 dpc | 39.3 | 33.2 | ***25.9*** | ***30.8*** | ***30.9*** |  | 34.0 | ***33.7*** | ***32.5*** | 37.9 | 37.3 |
| 6 dpc | 36.9 | 33.8 | ***24.7*** | ***26.8*** | nd |  | 34.5 | 37.3 | 33.6 | ***34.7*** | nd |
| 7 dpc | 30.5 | ***27.5*** | ***25.3*** | ***26.4*** | nd |  | 35.5 | 34.5 | 36.9 | 34.5 | nd |
| 8 dpc | 30.7 | ***21.5*** | ***27.5*** | 31.1 | nd |  | 37.0 | 35.9 | 37.0 | ***34.1*** | nd |
| 9 dpc | - | 33.5 | ***25.7*** | ***27.9*** | nd |  | - | - | 36.9 | 36.2 | nd |
| 10 dpc | - | 32.4 | nd | 32.7 | nd |  | 34.5 | - | nd | - | nd |
| 11 dpc | 40.9 | 36.3 | nd | 35.2 | nd |  | 38.9 | 42.0 | nd | - | nd |
| 12 dpc | 40.3 | 28.4 | nd | - | nd |  | - | 38.2 | nd | 37.8 | nd |
| 13 dpc | - | 27.7 | nd | 39.1 | nd |  | 37.9 | - | nd | - | nd |
| 14 dpc | - | 29.7 | nd | - | nd |  | - | - | nd | - | nd |

Group 2 contact ducks were mixed with group 1 at 1 dpi. Virus isolation positive swabs are indicated in bold italic. Swabs with undetectable Ct values (>45) were assigned a negative (-) value. Abbreviations: dpi (day post inoculation), dpc (day post contact), nd (not done, as ducks were euthanized for welfare reasons).
