# Supplementary material for: Experimentally Infected Domestic Ducks Show Efficient Transmission of Indonesian H5N1 Highly Pathogenic Avian Influenza Virus, but Lack Persistent Viral Shedding
Source: PLoS One. 2014 Jan 2;9(1):e83417. doi: 10.1371/journal.pone.0083417 (PMC3879242; doi:10.1371/journal.pone.0083417)
Supplement: Table S2 — Viral isolation and Ct rRT-PCR values for oral and cloacal swabs of the H5N1-inoculated ducks (group 3). (DOCX) [file pone.0083417.s002.docx]

**Table S2.** Viral isolation and Ct rRT-PCR values for oral and cloacal swabs of the H5N1-inoculated ducks (group 3).

| **Group 3 (inoculated ducks)** | | | | | | | | | | |
| --- | --- | --- | --- | --- | --- | --- | --- | --- | --- | --- |
| **Bird ID** | **#72** | **#76** | **#80** | **#81** | **#82** | **#90** | **#93** | **#97** | **#98** | **#99** |
| **Oral Swab** | | | | | | | | | | |
| Pre-Inoculation | - | - | - | - | - | - | - | - | - | - |
| 1 dpi | ***32.6*** | ***33.4*** | - | 37.1 | ***33.8*** | 37.6 | 32.5 | ***33.3*** | 35.4 | 35.3 |
| 2 dpi | ***30.5*** | 31.1 | - | 35.4 | 35.5 | 35.9 | 34.2 | ***29.9*** | 37.3 | 35.2 |
| 3 dpi | ***29.8*** | ***32.5*** | - | 30.3 | ***36.1*** | - | 35.2 | ***32.4*** | ***33.2*** | 33.3 |
| 4 dpi | ***29.7*** | 30.8 | - | ***29.8*** | 38.2 | - | ***34.1*** | ***30.5*** | 33.0 | 32.5 |
| 5 dpi | 32.3 | ***26.9*** | - | ***28.2*** | - | - | ***30.8*** | 33.2 | 35.6 | 28.9 |
| 6 dpi | 35.7 | ***29.5*** | - | 32.4 | 39.2 | 40.9 | 33.3 | 35.4 | 38.6 | 32.1 |
| 7 dpi | - | 34.1 | - | 34.3 | - | - | 37.2 | 37.0 | 34.1 | 34.3 |
| 8 dpi | - | 35.1 | 41.1 | 31.9 | - | - | - | 39.5 | - | 36.5 |
| 9 dpi | - | 35.8 | - | 36.6 | - | 43.1 | 42.5 | 40.8 | - | 40.5 |
| 10 dpi | - | - | 42.6 | 37.3 | - | - | - | - | - | - |
| 11 dpi | - | 43.1 | - | - | - | - | 38.9 | - | - | - |
| 12 dpi | - | 38.9 | - | - | 41.7 | - | - | 42.8 | - | 37.8 |
| 13 dpi | - | - | - | - | - | 38.7 | - | 38.3 | - | 38.5 |
| 14 dpi | - | - | - | - | - | - | 38.7 | - | - | 39.3 |
| 15 dpi | - | 38.7 | 38.0 | - | 38.3 | - | - | - | - | - |
| 17 dpi | - | - | - | - | - | - | - | - | - | - |
| 20 dpi | - | - | - | - | - | - | - | 41.4 | 41.3 | - |
| 24 dpi | 40.0 | - | - | - | - | - | - | - | - | - |
| 29 dpi | - | - | - | - | - | - | - | - | - | - |
| **Cloacal Swab** | | | | | | | | | | |
| Pre-Inoculation | - | - | - | - | - | - | - | - | - | - |
| 1 dpi | 33.6 | 31.7 | - | - | - | - | 38.9 | 35.8 | - | 41.0 |
| 2 dpi | 37.9 | 38.6 | - | - | 40.7 | - | - | 36.6 | - | 39.8 |
| 3 dpi | - | 38.8 | - | 39.2 | 41.7 | - | 38.8 | 39.4 | 43.4 | ***32.2*** |
| 4 dpi | - | 41.5 | - | 37.9 | - | - | 38.3 | 41.1 | - | ***34.2*** |
| 5 dpi | - | - | - | - | 39.1 | - | - | 36.2 | - | - |
| 6 dpi | 41.3 | - | 38.3 | - | 42.9 | 40.2 | - | - | 40.8 | 38.6 |
| 7 dpi | 39.6 | 39.7 | - | - | - | - |  | 38.1 | - | - |
| 8 dpi | - | - | - | - | - | - | - | - | - | - |
| 9 dpi | - | - | - | - | 41.0 | - | - | 40.0 | - | - |
| 10 dpi | - | - | - | - | - | - | - | - | - | - |
| 11 dpi | - | - | - | - | - | - | 41.3 | - | - | - |
| 12 dpi | - | - | 38.5 | 40.6 | - | - | - | - | - | - |
| 13 dpi | - | - | - | - | - | - | 36.2 | - | - | - |
| 14 dpi | - | - | - | - | - | 38.9 | - | - | 43.8 | 39.8 |
| 15 dpi | - | - | - | - | 39.4 | 39.3 | - | 38.1 | - | - |
| 17 dpi | - | - | - | - | 42.2 | - | - | - | - | - |
| 20 dpi | - | - | - | 38.5 | - | - | 44.9 | - | - | - |
| 24 dpi | - | - | - | - | - | - | - | - | - | 38.7 |
| 29 dpi | - | - | - | - | - | - | - | - | - | - |

Virus isolation positive swabs are indicated in bold italic. Swabs with undetectable Ct values (>45) were assigned as negative (-). For group 4 (not shown here), rRT-PCR was performed for selected oral and cloacal swabs collected from all the ducks at 1, 5 and 7 days post-contact after they were mixed with the inoculated ducks of group 3 at 10 dpi. Only five swabs from different contact ducks had detectable Ct values (38.0-42.6). Abbreviations: dpi (day post inoculation).
